# The origin of the ADAR gene family and animal RNA editing

**DOI:** 10.1186/s12862-015-0279-3

**Published:** 2015-01-29

**Authors:** Laura F Grice, Bernard M Degnan

**Affiliations:** School of Biological Sciences, University of Queensland, Brisbane, Queensland 4072 Australia

**Keywords:** A-to-I editing, Adenosine deaminase acting on RNA (ADAR), Evolution, RNA editing, Domain evolution, Sponge, Ctenophore, Metazoa, Domain architecture

## Abstract

**Background:**

ADAR (adenosine deaminase acting on RNA) proteins convert adenosine into inosine in double-stranded RNAs and have been shown to increase gene product diversity in a number of bilaterians, particularly mammals and flies. This enzyme family appears to have evolved from an ADAT (adenosine deaminase acting on tRNA) ancestor, via the addition of a double-stranded RNA binding domain. The modern vertebrate ADAR family is comprised of ADAD, ADAR2 and ADAR1, each of which has a conserved domain architecture. To reconstruct the origin of this protein family, we identified and categorised ADAR family members encoded in the genomes and/or transcriptomes of early-branching metazoan and closely related non-metazoan taxa, including thirteen sponge and ten ctenophore species.

**Results:**

We demonstrate that the ADAR protein family is a metazoan innovation, with the three ADAR subtypes being present in representatives of the earliest phyletic lineages of animals – sponges and ctenophores – but not in other closely related choanoflagellate and filasterean holozoans. *ADAR1* is missing from all ctenophore genomes and transcriptomes surveyed. Depending on the relationship of sponges and ctenophores to the rest of the Metazoa, this is consistent with either *ADAR1* being lost in ctenophores, as it has been in multiple metazoan lineages, or being an innovation that evolved after ctenophores diverged from the rest of the animal kingdom. The presence of Z-DNA binding domains in some sponge ADARs indicates an ancestral ADAR included this domain and it has been lost in multiple animal lineages.

**Conclusions:**

The ADAR family appears to be a metazoan innovation, with all family members in place in the earliest phyletic branches of the crown Metazoa. The presence of ADARs in sponges and ctenophores is consistent with A-to-I editing being a post-transcriptional regulatory mechanism that was used by the last common ancestor to all living animals and subsequently has been preserved in most modern lineages.

**Electronic supplementary material:**

The online version of this article (doi:10.1186/s12862-015-0279-3) contains supplementary material, which is available to authorized users.

## Background

RNA editing is a process of post-transcriptional RNA modification characterised by the insertion, deletion or modification of nucleotides [1,2]. One of the most prevalent forms of RNA editing is mediated by the ADAR (adenosine deaminase acting on RNA) class of editing molecules, that work both selectively and non-selectively to deaminate adenosine residues into inosines (A-to-I editing) in double-stranded RNA (dsRNA) substrates [3,4]. This editing can modify and regulate gene product output, for example via codon modification (as inosines are interpreted as guanosines by the cell), and influence splice site and small RNA functionality [5].

ADARs and A-to-I editing have been shown or proposed to play a role in diverse biological processes, the extent of which are not yet fully understood. Perhaps the best-studied role of ADARs is their involvement in editing neuronal receptor and ion channel components in taxa such as flies, squid and vertebrates [6]. ADARs have also been implicated in regulatory pathway roles, with suggested functions for A-to-I editing in RNAi antagonists [7], in pro- or antiviral mechanisms [8], and in the silencing of transposons and related sequences [9]. Gene-level regulation may also occur through editing-induced sequestration of transcripts within organelles [10] or modification of splice sites [11,12]. The primordial functionalities of the earliest ADAR systems are currently unknown.

ADATs (adenosine deaminase acting on tRNA) are critical proteins found in all eukaryotes. ADAT1 is equipped with a single adenosine deaminase (AD) domain, and is responsible for deamination of an adenosine in the tRNA wobble position into inosine [13]; ADAT1 does not play a role in RNA editing. ADARs appear to have originated via the incorporation of a double-stranded RNA binding (dsRB) domain-encoding region into the *ADAT1* coding sequence [13]. Duplication of this ancestral *ADAR* gene, and subsequent coding sequence and domain architecture diversification, has led to the generation of the ADAR family.

ADAR family members exist in bilaterians and cnidarians [14,15], and were recently identified in the genome of the ctenophore *Pleurobrachia bachei* [16]. They have not been found in the placozoan *Trichoplax adhaerens,* or in several non-metazoan eukaryotes, including choanoflagellates, fungi and plants, although these surveys have been limited in scope [14,15]. In this paper, we identify and categorise ADAR protein family members present in the earliest branching metazoan lineages, including thirteen sponge and ten ctenophore species, and thus conclude that the full, or nearly full, repertoire of ADAR protein family members existed in the last common ancestor to all contemporary animals.

## Results and discussion

### ADARs are present in the earliest branching metazoan lineages

**Figure 1:**
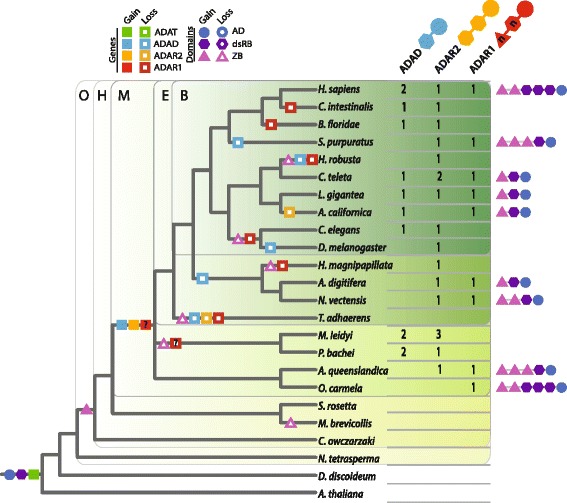
**Reconstruction of ADAR gene and domain evolution.** The table (right) lists the number of ADAR family members identified in each species. ADARs are classified based on their domain architecture, as shown by the ‘ball-and-stick’ protein models above each ADAR name. The Z-DNA/RNA binding (ZB) and double-stranded RNA binding (dsRB) domains of the ADAR1 model are marked with an ‘n’ to indicate that multiple copies of these domains may be present in different species. The domain architectures of all ADAR1-like proteins are depicted on the far right. The ADAR gene counts were used to reconstruct ADAT/ADAR evolution, as mapped to the phylogenetic tree as coloured squares (left). Searches for adenosine deaminase (AD), dsRB and ZB domains were performed to determine the phylogenetic positions of whole-genome domain origin and loss events, regardless of ADAT/ADAR complement; these events are also mapped to the tree as coloured shapes. Green boxes separate the tree into the main phylogenetic groupings: Bilateria (B), Eumetazoa (E), Metazoa (M), Holozoa (H) and Opisthokonta (O). For clarity, we present the sponge and ctenophore lineages on equal footing, and depict all three ADARs as present in the metazoan stem. The loss and gain of the ADAR1-like gene is marked with a question mark to illustrate the uncertainty in reconstructing these evolutionary events, which are elaborated upon further in Figure 3 and Additional file 2.

We identified ADARs in a number of key opisthokont and eukaryote taxa for which a draft genome is available. HMM and BLAST-based search methods were used to identify AD domain-encoding genes, and domain architecture predictions were employed to narrow this list to likely ADAR candidates (Additional file 1). ADAR sequences can be partitioned into three categories based on their overall domain architecture (Figure 1): ADAD-like (one dsRB domain and one AD domain); ADAR2-like (two dsRB and one AD domain); and ADAR1-like (any number of Z-DNA/RNA binding (ZB; z-alpha) and dsRB domains and one AD domain). These categories are based on *Homo sapiens* gene names and domain architectures. The *H. sapiens* ADAD sequence, while related to ADAR1 and ADAR2, is not implicated in RNA editing. ADAT-like sequences were identified in all species analysed (data not shown). We did not find evidence in invertebrates for ADAR3-like sequences, which possess an ADAR2-like architecture with an additional arginine-rich R-domain [17].

We identified novel candidate ADAR genes in the genomic sequences of representative species of two of the earliest-branching animal lineages – sponges (*Amphimedon queenslandica* and *Oscarella carmela*) and ctenophores (*Mnemiopsis leidyi*); our methodology also isolated the ADAR candidates recently reported from the ctenophore *Pleurobrachia bachei* [16]. We identified one each of an *ADAR1-* and *ADAR2-like* gene in *A. queenslandica*, a single *ADAR1-like* gene in *O. carmela*, and two *ADAD-* and three *ADAR2-like M. leidyi* genes (Figure 1). Of the previously identified *P. bachei* ADAR candidates [16], we categorised two sequences as *ADAD-like* and one as *ADAR2-like*, based on our domain architecture criteria (a comparison with candidates identified by Moroz et al. [16] is provided in Additional file 1). Analysis of the *Sycon ciliatum* unpublished genome reveals that this calcarean sponge possesses *ADAD-*, *ADAR2-* and *ADAR1-like* genes (Additional file 1). The presence of multiple ADAR types in sponges, ctenophores and other invertebrates is consistent with the idea that the metazoan last common ancestor was already equipped with a suite of ADARs comparable to the repertoire that exists in humans and other modern bilaterians, and that ADAR gene and domain loss occurred independently in multiple metazoan lineages (Figure 1).

### ADARs in the metazoan last common ancestor

**Figure 2:**
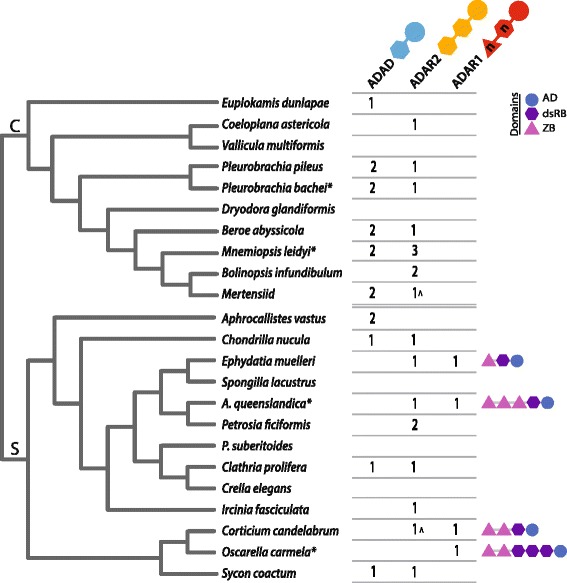
**ADAR family member distribution in sponges and ctenophores.** As in Figure 1, the number of candidate ADAR family members identified in each sponge and ctenophore genome (indicated by an asterisk) or transcriptome is shown. The domain architectures of ADAR1-like sequences are given on the far right. The phylogenetic relationships within the ctenophore (C, top) and sponge (S, bottom) lineages are depicted to the left. ADAR2 sequences indicated by a ^ are predicted to encode three dsRB domains. *Amphimedon queenslandica* and *Pseudospongosorites suberitoides* are abbreviated to conserve space.

Sponges and ctenophores are of significant evolutionary interest because they are considered the two earliest-branching metazoan lineages. However, questions remain as to whether sponges or ctenophores are the sister group to the rest of the Metazoa [18]. Although both taxa have multiple ADAR family members, all four examined species, *A. queenslandica, O. carmela, M. leidyi* and *P. bachei*, differ in their complement of ADAR genes. To facilitate a reconstruction of the evolution of the ADAR family, we searched for candidate ADAR sequences within the transcriptomes of an additional eleven sponge and eight ctenophore species (Figure 2; Additional file 1). Across the analysed sponge species, we identified candidate transcripts belonging to all three ADAR categories, *ADAD-, ADAR2-* and *ADAR1-like*. In no instance did a single species possess transcripts belonging to all three ADAR types (Figure 2); *ADAD-*, *ADAR2-* and *ADAR1-like* genes are however present in the *S. ciliatum* genome (Additional file 1). In ctenophores, no *ADAR1-like* transcripts were identified in any species; only *ADAD-* and *ADAR2-like* transcripts were identified, either together or separately. It should be noted, as these searches were performed on transcriptome data, that the failure to identify ADAR family members in particular species is not necessarily indicative that these sequences are absent from the genome; the overall lineage-specific trends do however allow insight into the taxonomic distribution of this protein family.

**Figure 3:**
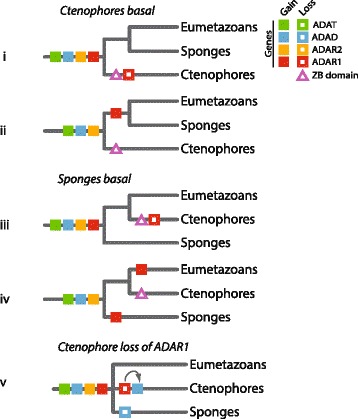
**Possible scenarios for ADAR evolution in the metazoan ancestor.** Five different scenarios of gene gain and loss events could explain the ADAR family distribution observed in sponges, ctenophores and eumetazoans, depending on whether sponges or ctenophores are the earliest-branching metazoan lineage. Filled and blank shapes represent gene (coloured squares) or ZB domain (triangles) gain and loss events, respectively. In panel v, the arrow represents the possible conversion of an ADAR1-like sequence to an ADAD-like architecture via domain loss.

Until the relative phyletic positions of sponges and ctenophores are fully resolved, multiple reconstructions of ADAR evolution are obtained depending if sponges or ctenophores are the earlier-branching phylum. ADAD-, ADAR2- and ADAR1-like proteins are all present in the sponge lineage, but ADAR1-like proteins, and indeed ZB domains entirely (data not shown), are absent in ctenophores. From this we conclude that ADAT-, ADAD- and ADAR2-like sequences were all present in the metazoan ancestor. ADAR1-like proteins were either present and subsequently lost in the ctenophore lineage, or gained later. If ctenophores branch first, the *ADAR1-like* gene was either lost in this taxon, along with the ZB domain (Figure 3, panel i) or gained in the sponge + eumetazoan clade after diverging from ctenophores (Figure 3, panel ii). Alternatively, if sponges are the most basal metazoans, the *ADAR1-like* gene was either lost in ctenophores (Figure 3, panel iii) or gained independently in both the sponge and eumetazoan groups (Figure 3, panel iv). Scenario iv appears to be less likely, as it would require *ADAR1-like* genes to evolve twice. A phylogenetic analysis of the ADAR family-associated AD domains from all analysed non-bilaterian genomes provided poor resolution regarding the evolutionary relationships between ADAD-, ADAR2 and ADAR1-like sequences (Additional files 2 and 3). However, as in earlier phylogenetic analyses of eumetazoan AD domains [15], the AD domains from non-bilaterian ADAR1-like sequences were found to form a cluster with reasonable bootstrap support, suggesting that the ADAR1-like gene has undergone little diversification across evolutionary history. Interestingly, the AD domain of an *M. leidyi* ADAD-like gene is also present in this ADAR1-like AD domain cluster (Additional file 2). This raises the possibility of a fifth evolutionary scenario of ADAR evolution (Figure 3, panel v) where the metazoan ancestor encoded all three ADAR family members, and that domain loss events converted a ctenophore ADAR1-like protein into a protein with ADAD-like architecture leaving ctenophores with two genes classifiable as ADAD-like. However, due to the poor bootstrap support for this tree overall, and as no *P. bachei* domain sequences are present in this cluster (Additional file 2), it is currently unclear whether this result is evolutionarily significant.

### Domain architecture of the ADAR1-like genes

*ADAR1-like* genes were identified in a diverse set of metazoans, and are present in a variety of domain conformations (Figures 1 and 2, far right). Human and other vertebrate *ADAR1* genes encode two ZB, three dsRB, and one AD domain, while the sea urchin *Strongylocentrotus purpuratus* genome encodes a protein equipped with three ZB, one dsRB and one AD domain. The *Nematostella vectensis* ADAR1 protein possesses two ZB (one of which is divergent), one dsRB and one AD domain. All ADAR1-like proteins identified in the other studied non-deuterostome eumetazoan taxa encode one copy each of the ZB, dsRB and AD domains. Interestingly, a diversity of domain architectures are encoded amongst the *ADAR1-like* genes and transcripts of sponges. In *A. queenslandica,* the *ADAR1-like* gene encodes three ZB, one dsRB and one AD domain, identical to the architecture of the *S. purpuratus* ADAR1, while the *O. carmela* gene encodes the vertebrate-like domain complement of two ZB, three dsRB and one AD domain (Figures 1 and 2); the unpublished *S. ciliatum* genome encodes an ADAR1-like protein with two ZB, one dsRB and one AD domain (Additional file 1). We also identified *ADAR1-like* transcripts from *Ephydatia muelleri* and *Corticium candelabrum*. These sequences both possess one dsRB and one AD domain, and the *E. muelleri* sequence contains one ZB domain while the *C. candelabrum* sequence has two (Figure 2).

The diversity of ADAR1-like architectures present in modern sponges complicates the resolution of the ancestral ADAR1-like form. However, a combination of one ZB, one dsRB and one AD domain remains the most parsimonious ancestral conformation; this form is seen within the sponge lineage (*E. muelleri*) and in all analysed non-deuterostome eumetazoan species except *N. vectensis*. ADAR1-like domain diversification has occurred in the sponge lineage, perhaps indicative of molecular tinkering allowing the testing and retaining in various species of different ADAR1-like domain architecture combinations. It is currently unknown whether similar levels of interspecies diversity exist in other phyla or classes.

### Origin of the metazoan ADAR protein family

*ADAT* genes are present throughout eukaryotes and are responsible for the deamination of adenosine into inosine for tRNA functionality [13]. Although AD and dsRB domains evolved prior to eukaryotic cladogenesis (Figure 1), the first evidence of these domains coming together to form an ancestral ADAR exists in the lineage leading to the crown Metazoa. This is likely to have occurred when a duplicated *ADAT* gene was coupled to a gene or part of a gene encoding one – or possibly more – dsRB domains, via domain shuffling. It appears most plausible that the first ADAR had one copy each of a dsRB and AD domain and thus was ADAD-like. This new gene then duplicated and incorporated a second dsRB domain, forming an *ADAR2-like* gene. The formation of the *ADAR1-like* gene involved the incorporation of one or more ZB domains into either an *ADAD-* or *ADAR2-like* gene. It is not clear which of these two family members was the original acceptor for the ZB domain, however, the combination of a single ZB and dsRB domain together in a number of species (Figures 1 and 2, far right) suggests the former is more likely. The ADAR suite was thus in place early in metazoan history. Minor alterations, namely gene loss and duplication events, have occurred in some animal lineages (Figures 1 and 2), but dramatic expansion and diversification events do not characterise the evolutionary history of the ADAR family.

## Conclusions

The ancestral role of the ADARs is currently unknown. Indeed, the biochemical functionality of basal metazoan ADAR protein family members in A-to-I editing remains to be tested experimentally. The existence of a diversified gene family in the earliest branching lineages of animals, but not in their close unicellular holozoan and fungal relatives, is consistent with this gene family being an animal-specific innovation. The evolution of metazoan multicellularity and complexity was accompanied by a wide range of genomic innovations [19]. The origin and expansion of the ADAR gene family prior to the diversification of crown metazoans is similar to other regulatory gene families, including microRNAs and piwiRNAs, and many transcription factor and signalling pathway families [20-22]. The maintenance of the ADAR gene family in most modern phyla suggests that RNA editing was and remains an essential part of the metazoan regulatory toolkit.

## Methods

### Identification of ADAR candidates from available draft genomes

HMMER 3.0 [23] was used to probe the unfiltered and filtered translated gene models from the genomes of each analysed species (Additional file 3) for AD domains [Pfam:PF02137] with a maximum Expect (E) value of 0.001. As confirmation, the *H. sapiens* ADAR1 protein sequence [Ensembl: ENST00000368474] was used as a query for reiterative PSI-BLAST searches against the NCBI refseq protein database for each species in turn [24], and also for BLAST searches in the genome browsers for each species. Domain architecture of the hits identified by each method was determined using Pfam [25], and sequences containing ADAR-associated domains (AD, dsRB [Pfam: PF00035] and ZB [Pfam: PF02295] domains) were selected. To be counted, each domain had a maximum E value of 0.001, however a small number of putative domains with higher E values were manually compared to the Pfam seed domain sequences; those deemed to be of sufficient similarity were included in subsequent analyses. Where identical, or very similar, sequences were identified using different search methods, the hit from the translated gene model dataset was used. Accession numbers and sequence sources are listed in Additional file 1.

### Identification of ADAR candidates from available sponge and ctenophore transcriptomes

Transcriptomes were downloaded and prepared as described in Additional file 3. Open reading frames were interrogated via hmmsearch and the domain architectures of resulting sequences were verified using Pfam, as for the genomic sequences above.

Sequence redundancies were observed in the transcriptomes of a number of species. To counter this, we partitioned sequences into groups sharing over 90% sequence identity, using the default parameters of the tool cd-hit [26], available via the CD-HIT Suite server [27]. We assigned the representative sequence from each cluster, as determined by cd-hit, to its relevant ADAR category. ADAR family member counts were mapped to a sponge-ctenophore phylogenetic tree [16,28]. Accession numbers of selected candidates are listed in Additional file 1.

### Availability of supporting data

The data sets supporting the results of this article are included within the article and its additional files.

## Additional files

Additional file 1:
**ADAR candidate information. Information regarding the sequences used for this study.** This excel file contains 4 sheets: 1. Analysed genes – Provides the accession number, source, domain architecture and additional comments regarding the identified ADAR candidates from analysed genomes. 2. Analysed transcripts – Provides the accession number, domain architecture and additional comments regarding the identified ADAR candidates from analysed sponge and ctenophore transcriptomes. 3. *P. bachei* comparison – Lists the putative ADAR and ADAT sequences previously identified by Moroz et al. [16] and cross-references these sequences to their categorisation in the present study, to avoid confusion due to similar nomenclature. 4. Novel sequences – Provides the domain architecture and sequences for unpublished ADAR candidates from *C. prolifera* (unpublished transcriptome), *S. ciliatum* (unpublished genome) and *O. carmela* (newly-generated Augustus gene models from publically-available data). Additional file 2:
**Phylogenetic analysis of adenosine deaminase domains.** Phylogenetic tree showing the relationship between AD domains from ADAD-, ADAR1- and ADAR2-like proteins. The tree was run with 1000 bootstrap replicates; bootstrap values greater than 500 are shown. While several branch points are not well supported, the ADAR1-like AD domains (and an additional ADAD-like sequence from *M. leidyi*) form a bootstrap-supported cluster. The *A. queenslandica* ADAT gene AD domain is included as an outgroup but was not explicitly designated as such for tree generation. Additional file 3:
**Supplementary Methods.** Provides additional methodological information regarding data sources, preparation of translated sequences for analysis, and generation of the phylogenetic tree given in Additional file 2.

## Abbreviations

AD: Adenosine deaminase [domain]
ADAR: Adenosine deaminase acting on RNA
ADAT: Adenosine deaminase acting on tRNA
dsRB: double-stranded RNA binding [domain]
dsRNA: double-stranded RNA
E: Expect [value]
ZB: Z-DNA/RNA binding [domain]
