# Supplementary material for: The origin of the ADAR gene family and animal RNA editing
Source: BMC Evol Biol. 2015 Jan 29;15(1):4. doi: 10.1186/s12862-015-0279-3 (PMC4323055; doi:10.1186/s12862-015-0279-3)
Supplement: Additional file 3: — Supplementary Methods. Provides additional methodological information regarding data sources, preparation of translated sequences for analysis, and generation of the phylogenetic tree given in Additional file 2. [file 12862_2015_279_MOESM3_ESM.docx]

**Additional File 3 – Supplementary Methods**

***Sources of sequence data***

We searched for ADAR candidates in the genomes of *Acropora digitifera* [1]*, Amphimedon queenslandica* [2]*, Aplysia californica* [3]*, Arabidopsis thaliana* [4]*, Branchiostoma floridae* [5]*, Caenorhabditis elegans* [6]*, Capitella teleta* [7]*, Capsaspora owczarzaki* [8]*,Ciona intestinalis* [9]*, Dictyostelium discoideum* [10]*, Drosophila melanogaster* [11]*, Helobdella robusta* [7]*, Hydra magnipapillata* [12]*, Lottia gigantea* [7]*, Mnemiopsis leidyi* [13]*, Monosiga brevicollis* [14]*, Nematostella vectensis* [15]*, Neurospora tetrasperma* [16]*, Oscarella carmela* [17]*, Pleurobrachia bachei* [18]*, Salpingoeca rosetta* [19]*, Strongylocentrotus purpuratus* [20], *Sycon ciliatum* (unpublished dataset, Maja Adamska and Marcin Adamski; details of analysed sequences are provided in Additional File 1) *and Trichoplax adhaerens* [21]. Transcriptome data was analysed from sponge species *Aphrocallistes vastus, Chondrilla nucula, Corticium candelabrum, Ircinia fasciculate, Petrosia ficiformis, Pseudospongosorites suberitoides, Spongilla lacustrus* and *Sycon coactum* [22], *Crella elegans* (non-reproductive tissue sample) [23], *Ephydatia muelleri* [24], and *Clathria prolifera* (unpublished dataset, Selene Fernandez-Valverde and Bernard Degnan; details of analysed transcripts are provided in Additional File 1).

***Preparation of translated sequences from sponge and ctenophore transcriptomes***

Gene models for *O. carmela* were predicted by submitting the whole genome assembly [17, 25, 26] to the Augustus v2.6.1 program [27]. Augustus was run using the *A. queenslandica* training set, with settings singlestrand=true, alternatives-from-evidence=true and uniqueGeneId=true; all other settings were run as default. Predicted amino acid sequences were extracted from the resulting file. Translated peptide sequences for *Ephydatia muelleri* were downloaded from Compagen [24, 25]. For remaining transcriptome datasets, the longest open reading frame between stop codons was determined for each sequence, using the program getorf available in the EMBOSS v6.5.7 software package [28].

***Generation of phylogenetic trees***

AD domain sequences from non-bilaterian ADAD-, ADAR1- and ADAR2-like sequences were used to generate a multiple sequence alignment, generated with 100 iterations of the built-in MUSCLE algorithm [29] in Geneious Pro 5.0.2 [30]. The *A. queenslandica* ADAT sequence Aqu1.212905 was also included as an outgroup. The alignment was manually refined in Geneious Pro, and submitted to the Gblocks webserver with the least stringent settings to further trim poorly-aligned regions [31, 32]. The ProtTest 2.4 webserver [33] was used to analyse the AD domain alignment and determine the best model selection method to use in generating phylogenetic trees, based on the AIC criterion. The best model was found to be LG+G. A maximum likelihood tree with 1000 bootstrap replicates was generated using the PhyML 3.0 webserver [34], with the SPR method of tree improvement and five random starting trees. The resulting tree was visualized in FigTree 1.4 [35] and aesthetic modifications were made during manuscript preparation.

**References**

1. Shinzato C, Shoguchi E, Kawashima T, Hamada M, Hisata K, Tanaka M, Fujie M, Fujiwara M, Koyanagi R, Ikuta T, Fujiyama A, Miller DJ, Satoh N: **Using the *Acropora digitifera* genome to understand coral responses to environmental change.** *Nature* 2011, **476**:320–323.

2. Srivastava M, Simakov O, Chapman J, Fahey B, Gauthier MEA, Mitros T, Richards GS, Conaco C, Dacre M, Hellsten U, Larroux C, Putnam NH, Stanke M, Adamska M, Darling A, Degnan SM, Oakley TH, Plachetzki DC, Zhai Y, Adamski M, Calcino A, Cummins SF, Goodstein DM, Harris C, Jackson DJ, Leys SP, Shu S, Woodcroft BJ, Vervoort M, Kosik KS, et al.: **The *Amphimedon queenslandica* genome and the evolution of animal complexity.** *Nature* 2010, **466**:720–726.

3. Broad Institute: **Aplysia Genome Project** [http://www.broadinstitute.org/science/projects/mammals-models/vertebrates-invertebrates/aplysia/aplysia-genome-sequencing-project]

4. The *Arabidopsis* Genome Initiative: **Analysis of the genome sequence of the flowering plant *Arabidopsis thaliana***. *Nature* 2000, **408**:796.

5. Putnam NH, Butts T, Ferrier DEK, Furlong RF, Hellsten U, Kawashima T, Robinson-Rechavi M, Shoguchi E, Terry A, Yu J-K, Benito-Gutiérrez EL, Dubchak I, Garcia-Fernàndez J, Gibson-Brown JJ, Grigoriev IV, Horton AC, de Jong PJ, Jurka J, Kapitonov VV, Kohara Y, Kuroki Y, Lindquist E, lucas S, Osoegawa K, Pennacchio LA, Salamov AA, Satou Y, Sauka-Spengler T, Schmutz J, Tadasuu S-I, et al.: **The amphioxus genome and the evolution of the chordate karyotype.** *Nature* 2008, **453**:1064–1071.

6. The C. elegans Sequencing Consortium: **Genome sequence of the nematode *C. elegans*: a platform for investigating biology.** *Science* 1998, **282**:2012–2018.

7. Simakov O, Marletaz F, Cho S-J, Edsinger-Gonzales E, Havlak P, Hellsten U, Kuo D-H, Larsson T, Lv J, Arendt D, Savage R, Osoegawa K, de Jong P, Grimwood J, Chapman JA, Shapiro H, Aerts A, Otillar RP, Terry AY, Boore JL, Grigoriev IV, Lindberg DR, Seaver EC, Weisblat DA, Putnam NH, Rokhsar DS: **Insights into bilaterian evolution from three spiralian genomes.** *Nature* 2013, **493**:526–531.

8. Suga H, Chen Z, de Mendoza A, Sebé-Pedrós A, Brown MW, Kramer E, Carr M, Kerner P, Vervoort M, Sánchez-Pons N, Torruella G, Derelle R, Manning G, Lang BF, Russ C, Haas BJ, Roger AJ, Nusbaum C, Ruiz-Trillo I: **The *Capsaspora* genome reveals a complex unicellular prehistory of animals**. *Nat Comms* 2013, **4**:1–9.

9. Dehal P, Satou Y, Campbell RK, Chapman J, Degnan B, De Tomaso A, Davidson B, Di Gregorio A, Gelpke M, Goodstein DM, Harafuji N, Hastings KEM, Ho I, Hotta K, Huang W, Kawashima T, Lemaire P, Martinez D, Meinertzhagen IA, Necula S, Nonaka M, Putnam N, Rash S, Saiga H, Satake M, Terry A, Yamada L, Wang H-G, Awazu S, Azumi K, et al.: **The draft genome of *Ciona intestinalis*: insights into chordate and vertebrate origins**. *Science* 2002, **298**:2157–2167.

10. Eichinger L, Pachebat JA, Glöckner G, Rajandream MA, Sucgang R, Berriman M, Song J, Olsen R, Szafranski K, Xu Q, Tunggal B, Kummerfeld S, Madera M, Konfortov BA, Rivero F, Bankier AT, Lehmann R, Hamlin N, Davies R, Gaudet P, Fey P, Pilcher K, Chen G, Saunders D, Sodergren E, Davis P, Kerhornou A, Nie X, Hall N, Anjard C, et al.: **The genome of the social amoeba *Dictyostelium discoideum***. *Nature* 2005, **435**:43–57.

11. Adams MD, Celniker SE, Holt RA, Evans CA, Gocayne JD, Amanatides PG, Scherer SE, Li PW, Hoskins RA, Galle RF, George RA, Lewis SE, Richards S, Ashburner M, Henderson SN, Sutton GG, Wortman JR, Yandell MD, Zhang Q, Chen LX, Brandon RC, Rogers YH, Blazej RG, Champe M, Pfeiffer BD, Wan KH, Doyle C, Baxter EG, Helt G, Nelson CR, et al.: **The genome sequence of *Drosophila melanogaster***. *Science* 2000, **287**:2185–2195.

12. Chapman JA, Kirkness EF, Simakov O, Hampson SE, Mitros T, Weinmaier T, Rattei T, Balasubramanian PG, Borman J, Busam D, Disbennett K, Pfannkoch C, Sumin N, Sutton GG, Viswanathan LD, Walenz B, Goodstein DM, Hellsten U, Kawashima T, Prochnik SE, Putnam NH, Shu S, Blumberg B, Dana CE, Gee L, Kibler DF, Law L, Lindgens D, Martinez DE, Peng J, et al.: **The dynamic genome of *Hydra***. *Nature* 2010, **464**:592–596.

13. Ryan JF, Pang K, Schnitzler CE, Nguyen A-D, Moreland RT, Simmons DK, Koch BJ, Francis WR, Havlak P, NISC Comparative Sequencing Program, Smith SA, Putnam NH, Haddock SHD, Dunn CW, Wolfsberg TG, Mullikin JC, Martindale MQ, Baxevanis AD: **The genome of the ctenophore *Mnemiopsis leidyi* and its implications for cell type evolution.** *Science* 2013, **342**:1242592.

14. King N, Westbrook MJ, Young SL, Kuo A, Abedin M, Chapman J, Fairclough S, Hellsten U, Isogai Y, Letunic I, Marr M, Pincus D, Putnam N, Rokas A, Wright KJ, Zuzow R, Dirks W, Good M, Goodstein D, Lemons D, Li W, Lyons JB, Morris A, Nichols S, Richter DJ, Salamov A, JGI Sequencing, Bork P, Lim WA, Manning G, et al.: **The genome of the choanoflagellate *Monosiga brevicollis* and the origin of metazoans.** *Nature* 2008, **451**:783–788.

15. Putnam NH, Srivastava M, Hellsten U, Dirks B, Chapman J, Salamov A, Terry A, Shapiro H, Lindquist E, Kapitonov VV, Jurka J, Genikhovich G, Grigoriev IV, Lucas SM, Steele RE, Finnerty JR, Technau U, Martindale MQ, Rokhsar DS: **Sea anemone genome reveals ancestral eumetazoan gene repertoire and genomic organization.** *Science* 2007, **317**:86–94.

16. Ellison CE, Stajich JE, Jacobson DJ, Natvig DO, Lapidus A, Foster B, Aerts A, Riley R, Lindquist EA, Grigoriev IV, Taylor JW: **Massive changes in genome architecture accompany the transition to self-fertility in the filamentous fungus *Neurospora tetrasperma***. *Genetics* 2011, **189**:55–69.

17. Nichols SA, Roberts BW, Richter DJ, Fairclough SR, King N: **Origin of metazoan cadherin diversity and the antiquity of the classical cadherin/β-catenin complex.** *Proc Natl Acad Sci USA* 2012, **109**:13046–13051.

18. Moroz LL, Kocot KM, Citarella MR, Dosung S, Norekian TP, Povolotskaya IS, Grigorenko AP, Dailey C, Berezikov E, Buckley KM, Ptitsyn A, Reshetov D, Mukherjee K, Moroz TP, Bobkova Y, Yu F, Kapitonov VV, Jurka J, Bobkov YV, Swore JJ, Girardo DO, Fodor A, Gusev F, Sanford R, Bruders R, Kittler E, Mills CE, Rast JP, Derelle R, Solovyev VV, et al.: **The ctenophore genome and the evolutionary origins of neural systems**. *Nature* 2014, **510**:109–114.

19. Fairclough SR, Chen Z, Kramer E, Zeng Q, Young S, Robertson HM, Begovic E, Richter DJ, Russ C, Westbrook MJ, Manning G, Lang BF, Haas B, Nusbaum C, King N: **Premetazoan genome evolution and the regulation of cell differentiation in the choanoflagellate *Salpingoeca rosetta***. *Genome Biol* 2013, **14**:R15.

20. Sea Urchin Genome Sequencing Consortium, Sodergren E, Weinstock GM, Davidson EH, Cameron RA, Gibbs RA, Angerer RC, Angerer LM, Arnone MI, Burgess DR, Burke RD, Coffman JA, Dean M, Elphick MR, Ettensohn CA, Foltz KR, Hamdoun A, Hynes RO, Klein WH, Marzluff W, McClay DR, Morris RL, Mushegian A, Rast JP, Smith LC, Thorndyke MC, Vacquier VD, Wessel GM, Wray G, Zhang L, et al.: **The Genome of the Sea Urchin *Strongylocentrotus purpuratus***. *Science* 2006, **314**:941–952.

21. Srivastava M, Begovic E, Chapman J, Putnam NH, Hellsten U, Kawashima T, Kuo A, Mitros T, Salamov A, Carpenter ML, Signorovitch AY, Moreno MA, Kamm K, Grimwood J, Schmutz J, Shapiro H, Grigoriev IV, Buss LW, Schierwater B, Dellaporta SL, Rokhsar DS: **The *Trichoplax* genome and the nature of placozoans.** *Nature* 2008, **454**:955–960.

22. Riesgo A, Andrade SC, Sharma PP, Novo M, Pérez-Porro AR, Vahtera V, González VL, Kawauchi GY, Giribet G: **Comparative description of ten transcriptomes of newly sequenced invertebrates and efficiency estimation of genomic sampling in non-model taxa**. *Front Zool* 2012, **9**:33.

23. Pérez-Porro AR, Navarro-Gómez D, Uriz MJ, Giribet G: **A NGS approach to the encrusting Mediterranean sponge *Crella elegans* (Porifera, Demospongiae, Poecilosclerida): transcriptome sequencing, characterization and overview of the gene expression along three life cycle stages**. *Mol Ecol Resour* 2013:n/a–n/a.

24. Richter DJ, Mora J, Nichols SA: ***Ephydatia muelleri* transcriptome assembly** [http://compagen.zoologie.uni-kiel.de/]

25. Hemmrich G, Bosch TCG: **Compagen, a comparative genomics platform for early branching metazoan animals, reveals early origins of genes regulating stem-cell differentiation.** *BioEssays* 2008, **30**:1010–1018.

26. Richter DJ, Mora J, Nichols SA: ***Oscarella carmela* transcriptome assembly** [http://compagen.zoologie.uni-kiel.de]

27. Stanke M, Tzvetkova A, Morgenstern B: **AUGUSTUS at EGASP: using EST, protein and genomic alignments for improved gene prediction in the human genome.** *Genome Biol* 2006, **7 Suppl 1**:S11.1–8.

28. Rice P, Longden I, Bleasby A: **EMBOSS: The European Molecular Biology Open Software Suite**. *Trends Genet* 2000, **16**:276–277.

29. Edgar RC: **MUSCLE: multiple sequence alignment with high accuracy and high throughput**. *Nucleic Acids Res* 2004, **32**:1792–1797.

30. Geneious Pro version 5.0.2 created by Biomatters. Available from http://www.geneious.com/

31. Castresana J: **Selection of conserved blocks from multiple alignments for their use in phylogenetic analysis.** *Mol Biol Evol* 2000, **17**:540–552.

32. Talavera D, Hospital A, Orozco M, la Cruz de X: **A procedure for identifying homologous alternative splicing events.** *BMC Bioinformatics* 2007, **8**:260.

33. Abascal F, Zardoya R, Posada D: **ProtTest: selection of best-fit models of protein evolution.** *Bioinformatics* 2005, **21**:2104–2105.

34. Guindon S, Dufayard J-F, Lefort V, Anisimova M, Hordijk W, Gascuel O: **New algorithms and methods to estimate maximum-likelihood phylogenies: assessing the performance of PhyML 3.0.** *Syst Biol* 2010, **59**:307–321.

35. Rambaut A: FigTree. [http://tree.bio.ed.ac.uk/software/figtree/]
